# Effect of ouabain on NFkB and p-38 activation in macrophages: a new biotechnological application

**DOI:** 10.1186/1753-6561-8-S4-P260

**Published:** 2014-10-01

**Authors:** Sandra Mascarenhas, Jacqueline Leite, Guilherme Galvão, Anne Alves

**Affiliations:** 1 Universidade Federal da Paraíba, Centro de Biotecnologia, Departamento de Biologia Celular Molecular, João Pessoa, Paraíba, Brazil; 2 Universidade Federal da Paraíba, Centro de Biotecnologia, Laboratório de Imunofarmacologia, João Pessoa, Paraíba, Brazil; 3 Universidade Federal da Paraíba, João Pessoa, Paraíba, Brazil

## Background

Inflammation is a response to external challenge or cellular injury that leads to the release of several inflammatory mediators and restoration of tissue structure and function. On the other hand, inflammation deregulation can cause tissue damage and is related to many diseases. During inflammation, a complex program of intracellular signal transduction and transcription events, driven by multiple pro-inflammatory mediators and cytokines, is activated. Ouabain, a potent inhibitor of the Na^+^, K^+^-ATPase, was identified as an endogenous substance of human plasma, able to affect various immunological processes, such as lymphocyte proliferation, apoptosis and monocyte function. These effects are particularly attractive for anti-inflamatory molecules bioprospection. We have also demonstrated the ability of ouabain to modulate inflammation, but little is known about the mechanisms involved.

## Aim

The aim of this work was to evaluate, *in vivo*, the role of ouabain on NFκB e p-38 activity. Additionally, we tested ouabain effect (1, 10 and 100 nM) on FITC-dextran endocytosis ability of peritoneal cells.

## Methods

In all experiments, 0.56mg/kg ouabain or phosphate buffered saline (PBS) was given intraperitoneally (i.p.) for three consecutive days. After 1h of the last day of ouabain treatment, mice were euthanized and macrophages and neutrophils from peritoneal cavity were collected. Cells were seeded in 24-well plates and incubated for 1h. After the incubation period, macrophages were tested using antibodies anti-P-NFκB e anti-Pp38 by flow cytometry. Besides that, ouabain was tested on FITC-dextran endocytosis ability of peritoneal cells. Peritoneal cells from unestimulated animals, including macrophages and neutrophils, were placed in sterile 96-well plates and incubated with different concentrations of ouabain (1, 10, 100nM) for 1h. This was followed by the addition of 0.5 mg/ml of 40 kDa FITC-dextran. After incubation, cells were washed with PBS and analyzed by flow cytometry. The FITC-dextran uptake was determined as the mean fluorescence relative to the background staining of the respective sample incubated with FITC-dextran at 4°C.

## Results

Ouabain treatment decreased basal levels of P-p38 and P-NFκB expression compared to the PBS group. Ouabain led to a 65% reduction of p38 activation and 60% reduction of P-NFκB. Besides that, ouabain was also capable of reducing by 84% the expression of both protein NFkB and p-38 in the double positive cell subpopulation. Finally, after one hour of incubation, approximately 58% of cells endocytosed FITC-dextran particles, and this process was inhibited at 4°C. Moreover, we also observed that ouabain did not affect the endocytosis of dextran particles.

## Conclusion

In agreement with the immunosuppressive effects that have been previously observed by us, ouabain pretreatment reduced the basal activation of proteins NFκB and p-38, but did not alter *in vitro *phagocytosis of dextran particles by peritoneal cells, suggesting a new mode of action of this substance. The present work reveals ouabain anti-inflammatory potential with biotechnological applications related to the immune system. However, further studies are necessary to elucidate the mechanisms involved.

## References

[B1] Harwood S Yaqoob MM Ouabain-induced cell signaling: Review Frontiers in Bioscience 2008 10 2011 17 15970473 10.2741/1676

[B2] De Vasconcelos DIB Leite JA Carneiro LT Piuvezam MR De Lima MRV De morais LCL Rumjanek VM Rodrigues-Mascarenhas S Anti-inflammatory and antinociceptive activity of ouabain in mice Mediat Inflamm 2011 912 25 10.1155/2011/912925 PMC3136139 21772669

[B3] Blaustein MP Zhang J Chen L Song H Raina H Kinsey SP Izuka M Iwamoto T Kotlikoff MI Lingrel JB Philipson KD Wier WG Hamlyn JM The pump, the exchanger, and endogenous ouabain Hypertension 2009 53 291 98 10.1161/HYPERTENSIONAHA.108.119974 19104005 PMC2727927

[B4] Hallett JM Leitch AE Riley NA Duffin R Haslett C Rossi AG Novel pharmacological strategies for driving inflammatory cell apoptosis and enhancing the resolution of inflammation Trends Pharmacol Sci 2008 29 250 57 10.1016/j.tips.2008.03.002 18407359

